# The mediating effect of clinical belongingness on the relationship between anxiety and professional identity in nursing interns: a cross-sectional study

**DOI:** 10.3389/fmed.2024.1489383

**Published:** 2025-01-15

**Authors:** Junhao Zhang, Lijia Wang, Xue Yang, Yuwei Yang, Xuehua Wu, Huaping Huang, Guirong Li

**Affiliations:** ^1^School of Nursing, Chengdu Medical College, Chengdu, Sichuan, China; ^2^Department of Clinical Laboratory, Mianyang Central Hospital, School of Medicine, University of Electronic Science and Technology of China, Mianyang, Sichuan, China; ^3^School of Nursing, Sichuan College of Traditional Chinese Medicine, Mianyang, Sichuan, China; ^4^Nursing Department of Mianyang Central Hospital, School of Medicine, University of Electronic Science and Technology of China, Mianyang, Sichuan, China

**Keywords:** anxiety, clinical belongingness, professional identity, mediating effect, nursing interns

## Abstract

**Background:**

Previous studies have reported that anxiety negatively affects professional identity (PI), and clinical belongingness is positively correlated with PI among nursing interns. However, little is known about the relationship between anxiety, PI, and clinical belongingness among nursing interns.

**Objective:**

To explore the relationship between PI, clinical belongingness, and anxiety among nursing interns, and to demonstrate the mediating role of clinical belongingness in this relationship.

**Methods:**

A cross-sectional study using an online questionnaire was conducted between November 2023 and January 2024 among 1,050 nursing interns from 26 teaching hospitals in Sichuan Province, China. A general information questionnaire, the Belongingness Scale-Clinical Placement Experience (BES-CPE), the Self-Rated Anxiety Scale (SAS), and the Professional Identity Questionnaire for Nursing Students (PIQNS) were used to collect data. SPSS (version 25.0) and AMOS (version 28.0) were used to analyze the data.

**Results:**

Nursing interns had moderate mean scores for all variables; PI (60.77 ± 12.18), clinical belongingness (115.99 ± 17.02), and anxiety (47.44 ± 8.75). Correlation analyses revealed that both PI (*r* = −0.129, *p* < 0.01) and clinical belongingness (*r* = −0.087, *p* < 0.01) were negatively correlated with anxiety and clinical belongingness was positively correlated with PI (*r* = 0.601, *p* < 0.01). The clinical belongingness of nursing interns had a mediating effect on the relationship between anxiety and PI (*β* = −0.072, 95% confidence interval = −0.133 to −0.013, *p* < 0.001), accounting for 40% of the total effect.

**Conclusion:**

The anxiety level of nursing interns can have a direct impact on the prediction of PI and an indirect influence on PI mediated by clinical belongingness. Accordingly, nursing educators and managers should screen and channel the mental health problems of nursing interns in a timely manner, improving their clinical belonging, which will help improve PI and ultimately improve the stability of the nursing workforce.

## 1 Introduction

The shortage of nurses has become an important issue in global health systems (1). In particular, aging populations, international conflicts, the climate crisis, and financial instability have exacerbated the imbalance in medical and health resources (2, 3). According to the World Health Organization estimate, if no interventions are implemented, there will be a global nursing shortage of 5.7 million by 2030 (4). The considerable shortage of nursing staff restricts the growth of the nursing workforce and affects the quality of nursing care (5). Nursing interns are a new force in the future development of nursing careers, and professional identity (PI) plays an important role in the quality training of modern nursing talent (6, 7).

PI is a positive assessment of the cognitive and emotional perceptions of nursing interns with respect to clinical nursing work and the nursing profession, which directly affects their willingness to participate in nursing work in the future and the stability of nursing teams (8). Furthermore, Li et al. found that a strong PI not only improves the professional confidence of nursing interns and clinical belongingness but also minimizes the accumulation of negative emotions, such as anxiety, depression, and burnout (9). However, influenced by traditional culture, social environment, and working environment, nursing interns tend to have a lower sense of PI (10). A systematic review found that low PI awareness was the leading cause of burnout and turnover among nursing interns (11). It should be noted that anxiety is common in nursing interns. Before entering a hospital, nursing interns typically have a romantic perception of the nursing profession, as nursing involves caring for and treating patients. However, in reality, high standards requirements, heavy workloads, low salary, and poor working conditions can cause various psychological problems for nursing interns, particularly anxiety (12). Studies have shown that anxiety in nursing interns has a negative effect on their PI (13). High levels of anxiety not only affect the PI of nursing interns, but also have long-term negative consequences on their willingness to become nurses, affecting career planning, and causing serious mental health problems (14). Furthermore, nursing interns experience more severe anxiety than students in other medical disciplines, which can persist throughout their clinical practice (15). Therefore, more emphasis must be placed on the relationship between anxiety and PI among nursing interns to strengthen their intention to remain in the nursing field.

The concept of clinical belongingness refers to nursing interns in clinical practice who feel accepted, recognized, respected, and appreciated (16). According to the Maslow hierarchy of needs, belongingness and love are fundamental human needs (17). Therefore, some relevant studies have been conducted on the relationship between clinical belongingness and PI among nursing interns and it was found that clinical belongingness was positively correlated with PI (18). Clinical belongingness is essential for nursing interns to feel motivated to learn, to actively participate in clinical practice, to increase professional confidence, and develop PI (19). In addition, studies have suggested that nursing interns with a high level of clinical belongingness are more likely to have better interpersonal relationships, a better sense of adaptive skills to overcome complex clinical problems, and be more capable of coping with nervousness and anxiety (20, 21). Consequently, it is hypothesized that clinical belongingness plays a positive moderating role in the relationship between anxiety and PI.

**Figure 1:**
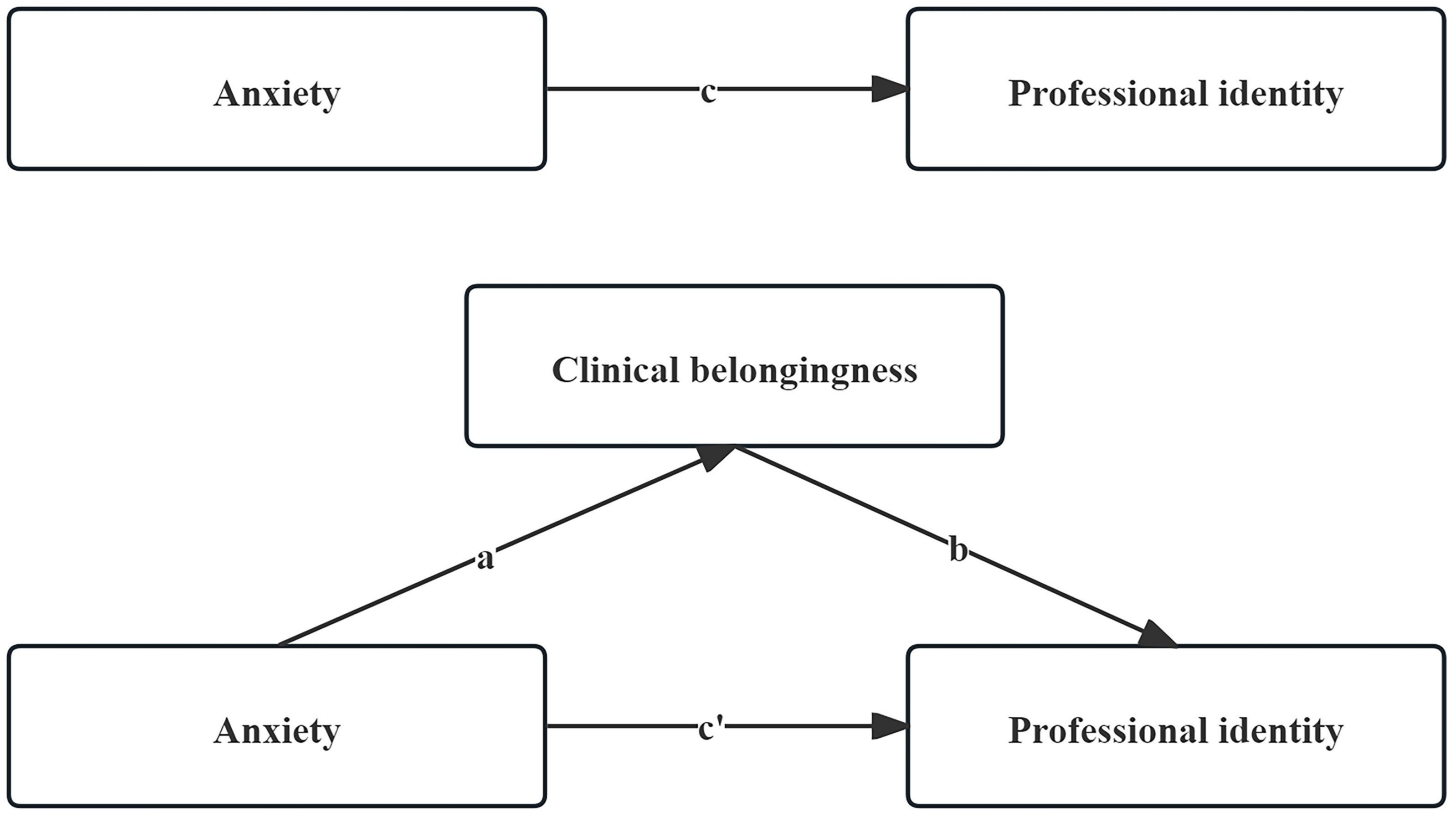
The conceptual framework diagram of this study.

Previous studies have found that clinical belongingness among nursing interns is positively correlated with PI and that anxiety has a negative effect on PI (13, 18). However, most previous research has focused on the impact of professional identity on nursing interns, few studies have reported the relationship between PI, clinical belongingness, and anxiety among nursing interns; and there are fewer researches on the specific mechanism of the action between these three variables and the construction of a model using them. Therefore, this study focuses on a group of nursing interns as research objects to explore the intrinsic mechanisms and mediating effects of clinical belongingness, PI, and anxiety among them. Based on the above findings, it is further hypothesized that clinical belongingness plays a positive moderating role in the relationship between anxiety and PI (Figure 1), the following hypotheses about the nursing interns are formulated:

*H1*: PI is positively correlated with clinical belongingness.

*H2*: Clinical belongingness is negatively correlated with anxiety.

*H3*: PI is negatively correlated with anxiety.

*H4*: Clinical belongingness plays a mediating role between PI and anxiety.

## 2 Methods

### 2.1 Study participants

A cross-sectional study was conducted between November 2023 and January 2024 using an anonymous self-administered online questionnaire in 26 teaching hospitals from the members of the Nursing Professional Committee of Sichuan Province, including 18 tertiary hospitals and 8 secondary hospitals. The convenience sampling was chosen due to its practicality and feasibility. Inclusion criteria were: (1) nursing interns, including secondary education, college, undergraduate, or postgraduate; (2) clinical practice for more than three months; and (3) signed informed consent and voluntary participation. The exclusion criterion was absence during the internship period of more than one month. Based on the Kendall (22) sample size estimation method, the recommended sample size for descriptive research should be 10 to 20 times the number of variables. In this study, there were 10 variables from the sociodemographic characteristics questionnaire and 12 variables from 3 scales, totaling 22 items. Considering that 20% of the questionnaires were invalid, the sample size was estimated to be 264 to 528. However, to achieve more accurate and stable results, a larger sample sizes was recruited. Ultimately, this process yielded a final sample of 1,050 valid responses from a total of 1,271 nursing interns. Of these, 221 questionnaires were excluded: 59 due to high response homogeneity across all items, 30 due to logical inconsistencies across the items, and 132 for having completion times of <3 minutes, with a valid recovery rate of 82.6%.

### 2.2 Measurement

#### 2.2.1 General information questionnaire

A self-designed general information questionnaire was used to investigate gender, age, duration of the completed internship, educational level, place of origin, grades of the internship hospital, whether it was an only child, whether they chose the nursing profession voluntarily, student leadership experience and having anyone in the medical profession in the family.

#### 2.2.2 Belongingness scale-clinical placement experience (BES-CPE)

BES-CPE was developed by Levett-Jones et al. (23) and adapted by Tian et al. (24) for Chinese culture to assess the level of clinical belongingness among medical professionals. The scale consists of 31 items, of which items 9, 12, and 23 were reverse-scored. Dimensions 1–3 represent self-esteem, communication, and efficacy, respectively. A five-point Likert scale was used (1, never true; 2, rarely true; 3, occasionally true; 4, frequently true; and 5, always true). Higher scores indicate higher levels of clinical belongingness. The Cronbach’s *α* coefficient of reliability of the scale was 0.96.

#### 2.2.3 Professional identity questionnaire for nurse students (PIQNS)

The PIQNS is widely used to measure the PI of nursing students in China (25). The 17-item scale is categorized into five dimensions: professional self-image, retention benefits and exit risks, social comparison, self-reflection, independence of career choice, and social modeling. Each item was rated on a five-point Likert scale, ranging from 5, strongly agree; to 1, never agree; of which item 12 was reverse-scored. The higher the score, the more positive the PI. The scale had good reliability and validity, with a Cronbach’s *α* coefficient of 0.95.

#### 2.2.4 Self-rating anxiety scale (SAS)

The SAS was developed by Zung (26), with four dimensions and 20 items, of which, questions 5,9,13,17, and 19 were reverse scored. A four-point scale ranging from 1, never to 4, always was used. The SAS standard score was calculated by summing all items, multiplying by 1.25, and rounding to an integer. The thresholds for identifying anxiety symptoms were defined as a standard score of <50 (no symptoms), 50–59 (mild anxiety), 60–69 (moderate anxiety) or 70 (severe anxiety). The Cronbach’s *α* coefficient was 0.81.

### 2.3 Quality control

All questionnaires were imported into the online platform, WENJUANXING.^1^ To ensure data quality, we conducted pilot tests before hand to estimate the completion time and make the necessary adjustments. The system limited each account or IP address to only one submission, and if the data were incomplete or missing, participants were unable to submit their questionnaires through the survey platform. Based on the agreement of the nursing education team leaders of 26 teaching hospitals, questionnaires were distributed to nursing interns who met the inclusion and exclusion criteria. The purpose and content of this study were explained, and informed consent was obtained before the questionnaire was distributed. In addition, the nursing interns who participated in this study did not receive any reward after completing the questionnaire. Questionnaires completed in less than 3 minutes or showing patterns of regularity, consistency (80% of the answers were the same option), or logical inconsistencies were excluded.

### 2.4 Data analyses

All data were analyzed using SPSS 25.0. Categorical variables were described by numbers with percentages, and inter-group comparison was carried out by the chi-square test. Continuous variables that conform to a normal distribution were expressed as means with standard deviation (SD), and inter-group comparison was performed by using independent t-test and the ANOVA test. Pearson’s correlation analysis was used to explore the correlations between PI, clinical belongingness, and anxiety. AMOS 28.0 was used for structural equation modeling (SEM). An adequate model is considered acceptable if the threshold values are as follows: χ^2^/df ≤ 5.00, RMSEA <0.08, and CFI/GFI/NFI/IFI/AGFI > 0.90 (27, 28). Subsequently, a stepwise regression analysis investigated associations between anxiety and PI, and the potential mediating role of clinical belongingness in these associations was explored. Additionally, the mediation test was performed using the SPSS PROCESS macro to validate the data and calculate the 95% confidence intervals (CIs). *p* < 0.05 was considered statistically significant.

## 3 Results

### 3.1 Sociodemographic characteristics of nursing interns

Table 1 shows the baseline characteristics of the nursing interns. A total of 1,050 questionnaires were collected with an effective recovery rate of 94.94%. Of these, 82.5% were female, 28.9% were the one-child family, 74.5% were from rural areas, 59.6% had experience as a student leader, 25.2% voluntarily chose the nursing profession, and 25.6% of family members or relatives were medical workers.

**Table 1:** Sociodemographic characteristics of nursing interns (*N* = 1,050).

Demographics		*N* (%)	PI (M ± SD)	CB (M ± SD)	Anxiety (M ± SD)
Gender	Female	866 (82.5%)	60.23 ± 11.99	115.44 ± 16.89	44.34 ± 7.54
	Male	184 (17.5%)	63.27 ± 12.79	118.56 ± 17.43	48.10 ± 8.85
	*t*		3.09	2.26	−5.36
	*P*		**<0.01**	**0.02**	**<0.01**
Age (years)	**<**18	160 (15.2%)	64.49 ± 11.08	116.38 ± 17.76	46.83 ± 9.17
	18–20	420 (40.0%)	61.72 ± 11.95	115.94 ± 17.53	47.57 ± 9.14
	21–23	396 (37.7%)	58.94 ± 12.49	115.91 ± 16.42	47.41 ± 8.14
	>23	74 (7.0%)	57.05 ± 11.62	115.84 ± 15.84	48.21 ± 8.84
	*F*		11.42	0.03	0.48
	*P*		**<0.01**	0.99	0.70
Duration of internship (months)	3	15 (1.4%)	57.60 ± 9.99	110.53 ± 13.11	49.08 ± 8.92
	4–9	937 (89.2%)	60.89 ± 12.06	115.97 ± 17.00	47.39 ± 8.54
	>10	98 (9.3%)	60.05 ± 13.58	116.94 ± 17.64	47.65 ± 10.61
	*F*		0.72	0.92	0.31
	*P*		0.49	0.40	0.74
Education level	Technical secondary school	235 (22.4%)	64.08 ± 11.75	116.46 ± 17.97	46.52 ± 8.94
	Junior college	522 (49.7%)	62.06 ± 12.04	116.50 ± 17.12	47.47 ± 8.85
	Bachelor	219 (20.9%)	55.96 ± 11.37	114.76 ± 16.35	48.29 ± 8.26
	Master	74 (7.0%)	55.31 ± 11.12	114.50 ± 15.05	47.60 ± 8.81
	*F*		25.75	0.79	1.57
	*P*		**<0.01**	0.50	0.20
Place of origin	Urban	268 (25.5%)	59.69 ± 12.60	116.25 ± 16.97	47.63 ± 8.81
	Rural	782 (74.5%)	61.14 ± 12.02	115.90 ± 17.04	46.88 ± 8.58
	*t*		1.68	−0.29	1.22
	*P*		0.09	0.77	0.22
Grades of Internship hospital	Level II	107 (10.2%)	61.36 ± 12.99	113.15 ± 16.76	45.77 ± 7.67
	Level III	943 (89.8%)	60.70 ± 12.09	116.31 ± 17.03	47.63 ± 8.85
	*t*		0.54	−1.82	−2.08
	*P*		0.59	0.07	**0.04**
Whether the only child	Yes	303 (28.9%)	61.88 ± 13.15	117.55 ± 17.68	47.39 ± 8.70
	No	747 (71.1%)	60.31 ± 11.75	115.35 ± 16.71	47.56 ± 8.89
	*t*		−1.80	−1.90	−0.28
	*P*		0.07	0.06	0.78
Whether chose nursing profession voluntarily	Yes	787 (75.0%)	63.69 ± 11.58	118.33 ± 17.04	48.75 ± 8.69
	No	263 (25.0%)	52.02 ± 9.47	108.96 ± 14.91	47.00 ± 8.73
	*t*		−16.32	−7.96	2.80
	*P*		**<0.01**	**<0.01**	**0.01**
Student leadership experience	Yes	626 (59.6%)	61.51 ± 12.06	117.23 ± 16.81	47.49 ± 8.77
	No	424 (40.4%)	59.67 ± 12.30	114.15 ± 17.18	47.41 ± 8.75
	*t*		−2.41	−2.89	0.15
	*P*		**0.02**	**<0.01**	0.88
Anyone in the medical profession in the family	Yes	269 (25.6%)	62.55 ± 12.22	118.31 ± 17.94	47.38 ± 8.62
	No	781 (74.4%)	60.15 ± 12.12	115.19 ± 16.62	47.61 ± 9.14
	*t*		−2.80	−2.60	−0.36
	*P*		**0.01**	**0.01**	0.72

CB, clinical belongingness; PI, professional identity. The bold values are intended to highlight general data with *P* < 0.05 and can be represented routinely.

### 3.2 The mean scores for clinical belongingness, PI, and anxiety of nursing interns

The mean scores for clinical belongingness, PI and anxiety were 115.99 ± 17.02, 60.77 ± 12.18, and 47.44 ± 8.75, respectively (Table 2). The detection rate of anxiety was 35.52% (677/1050). Of those, 26.86% (282/1050), 6.10% (64/1050), and 2.57% (27/1010) of the nursing interns had mild, moderate, or severe anxiety, respectively.

**Table 2:** The overall scores of the CB, PI and anxiety scale of nursing interns (*N* = 1,050).

Variables	Items	Total score (M ± SD)	Item score (M ± SD)
CB	31	115.99 ± 17.02	3.74 ± 0.55
Self-esteem	13	47.31 ± 6.78	3.64 ± 0.52
Communication	10	36.88 ± 6.26	3.69 ± 0.63
Efficacy	8	31.80 ± 4.80	3.97 ± 0.60
PI	17	60.77 ± 12.18	3.57 ± 0.72
Social modeling	2	7.59 ± 1.78	3.79 ± 0.89
Independence of career choice	2	6.64 ± 1.47	3.32 ± 0.73
Social comparison and self-reflection	3	11.43 ± 2.05	3.81 ± 0.68
Retention benefits and exit risks	4	13.65 ± 3.42	3.41 ± 0.85
Professional self-image	6	21.45 ± 5.14	3.58 ± 0.86
Anxiety	20	47.44 ± 8.75	1.90 ± 0.35
Anxious mood	4	6.86 ± 2.60	1.72 ± 0.65
Motor nervous	6	12.52 ± 2.32	2.09 ± 0.39
Autonomic system disturbance	8	14.57 ± 3.31	1.82 ± 0.41
Mixed factor	2	4.00 ± 1.31	2.00 ± 0.66

CB, clinical belongingness; PI, professional identity.

### 3.3 Correlation analyses

Table 3 presents a detailed overview of the relationships between the variables. The clinical belongingness of the nursing interns was negatively correlated with anxiety (*r* = −0.087, *p* < 0.01), positively correlated with PI (*r* = 0.601, *p* < 0.01), therefore, H1 and H2 were supported. Meanwhile, and there was a negative correlation between anxiety and PI (*r* = −0.129, *p* < 0.01). The result indicated support for H3.

**Table 3:** Pearson’s correlation analysis of CB, PI, and anxiety.

Variables	CB	Anxiety	PI
CB	1		
Anxiety	−0.087**	1	
PI	0.601**	−0.129**	1

^**^p < 0.01. CB, clinical belongingness; PI, professional identity.

### 3.4 Analysis of mediating effects

A stepwise regression analysis investigated the association between anxiety and PI, and the potential mediating role of clinical belongingness in these associations was explored. PI as a dependent variable (y), anxiety as an independent variable (x), and clinical belongingness as a mediating variable (m). In step 1, PI and anxiety were included into the regression equation, and it showed that anxiety had a negative predictive effect on PI (*β* = −0.129, *p* < 0.001). In step 2, clinical belongingness and anxiety were included into the regression equation, and the result showed that anxiety also had a negative predictive effect on clinical belongingness (*β* = −0.087, *p* < 0.01). In step 3, clinical belongingness, PI, and anxiety were included into the regression equation, and the result was that anxiety had a negative predictive effect on PI (*β* = −0.078, *p* < 0.01). However, clinical belongingness had a positive predictive effect on professional identity (*β* = 0.594, *p* < 0.001) (Table 4).

**Table 4:** The regression results of the effects of PI and anxiety on CB.

Step	Outcome variable	Predictor	*β*	*t*	*R*	*R* ^2^	*F*
1	PI	Anxiety	−0.129	−4.219***	0.129	0.017	17.802
2	CB	Anxiety	−0.087	−2.829**	0.087	0.008	8.002
3	PI	Anxiety	−0.078	−3.141**	0.606	0.367	303.839
		CB	0.594	24.084***			

^**^p < 0.01, ^***^p < 0.001. CB, clinical belongingness; PI, professional identity.

### 3.5 Verifying the hypothesized model

Under the condition of controlling gender, age, education level, duration of internship, place of origin, grades of internship hospital, whether the only child, whether chose nursing profession voluntarily, student leadership experience, anyone in the medical profession in the family. The mediating effect of clinical belongingness on anxiety and PI in nursing interns was analyzed. This study showed that the indirect effect was statistically significant and represented 40% of the total effect, with a value of −0.072 (95%Cl: [−0.133, −0.013], *p* < 0.05); the direct effect was statistically significant and represented 60% of the total effect, with a value of −0.108 (95%Cl: [−0.192, −0.030], *p* < 0.05), respectively (Table 5).

**Table 5:** Direct and indirect effects of clinical belongingness.

	Effect	SE	95%CI		%
			LLCL	ULCL	
Indirect effect	−0.072	0.031	−0.133	−0.013	40%
Direct effect	−0.108	0.042	−0.192	−0.030	60%
Total effect	−0.180	0.045	−0.199	−0.022	-

SE, Standard Error; CI, Confidence Interval; LLCI, Lower Limit Confidence Interval; ULCL, Upper Limit Confidence Interval.

**Figure 2:**
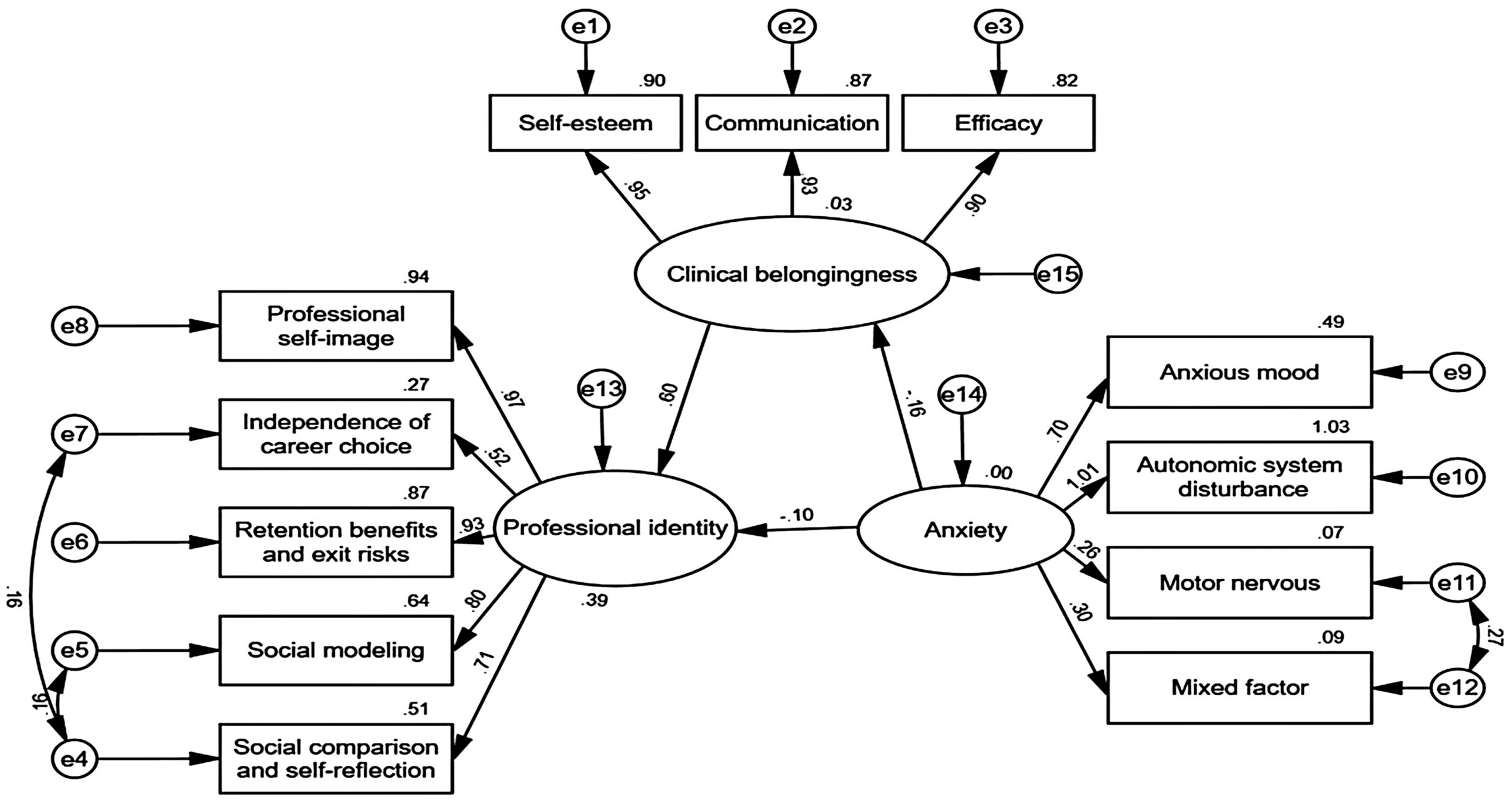
Final model and standardized model paths.

Given that the χ^2^/df ratio is significantly influenced by sample size and the sample size in this study was 1,050, this metric should not be used to reject the model (29). The large sample size in this study contributed to the general robustness of the findings. The successfully fitted model is illustrated in Figure 2. According to Harman’s single-factor test, there were nine factors with an eigenvalue >1, and the first factor explained 33.51% of the variation, which is lower than the critical value of 40.0%. Thus, the data did not have serious common method bias. The model fitting index of the model is acceptable (χ^2^/df = 6.765, RMSEA = 0.072, CFI = 0.938, GFI = 0.959, NFI = 0.969) (Table 6). This finding indicates that clinical belongingness significantly mediates the relationship between anxiety and PI among nursing interns. Therefore, H4 was supported. The effects of the interactions between the variables in the model are visually represented in Figure 1 and Table 5.

**Table 6:** Summary of model fit indices.

Model	χ^2^/df	RMSEA	GFI	AGFI	NFI	IFI	CFI
Recommended value	≤5	≤0.08	≥0.90	≥0.90	≥0.90	≥0.90	≥0.90
Actual value	6.765	0.072	0.959	0.930	0.969	0.974	0.974

RMSEA, root mean square error of approximation; GFI, goodness-of-fit index; NFI, normed fit index; IFI, incremental fit index; CFI, comparative fit index.

## 4 Discussion

This study explored the relationships between PI, clinical belongingness, and anxiety among nursing interns. These results are consistent with the proposed conceptual research model. First, the results showed that the anxiety of nursing interns was negatively correlated with clinical belongingness and PI, while clinical belongingness was positively correlated with PI. Second, the mediating effect showed that clinical belongingness had a partial mediating effect between anxiety and PI of nursing interns.

### 4.1 Analysis of the current status of PI in nursing interns

In this study, the mean PI score was 60.77 ± 12.18, which was at the medium level and consistent with the findings of other studies (30, 31). Nursing interns with various characteristics showed obvious differences in their PI scores (32). Nursing interns with a college degree or lower had a higher PI than those with a bachelor’s degree or higher. This could be attributed to the increased educational output of nursing interns as well as their higher expectations of professional treatment and standing. However, the current nursing employment environment was not positively correlated with degree level (33). With the expansion of the graduate nursing program, competition among graduates is increasing, leading to employment and career pressure increased significantly (34). Additionally, in a busy clinical work environment, with a low salary and influenced by the traditional Chinese impression, the public image of nurses is viewed as performing simple and repetitive tasks and having to follow the orders of doctors, which leads to nurses often being overlooked by people and low PI (35). As a result of the combination of these factors, some nursing interns have to reconsider their future career paths.

The results of this study showed that the majority of nursing interns chose nursing majors voluntarily, and compared to involuntary nursing interns, they had higher PI and clinical belongingness, and a lower rate of anxiety. This is because the nursing interns who voluntarily enrolled in a nursing major were already familiar with, and understood, the nursing major before choosing it. Consequently, they were able to more effectively formulate their career plans and respond positively to academic challenges (36). Furthermore, the PI of male nursing interns was higher than that of female nursing interns, contrary to the results of previous studies (37), which may be related to the increasing social demand for male nurses and the stereotype of nursing as being a profession only for females, which is gradually disappearing (38). However, there were fewer male nursing interns in this survey; therefore, the results may have been biased.

Furthermore, PI is a dynamic developmental process, which means that the PI of nursing interns can be reconstructed or strengthened (39). Therefore, to improve the PI of nursing interns, nurse educators and clinical practice managers should pay attention to the PI of nursing interns with different characteristics, strengthen the construction of a professional culture, assist nursing interns to better integrate into the group, formulate a personalized internship plan, and provide a clear career plan.

### 4.2 Correlation analysis of PI, clinical belongingness, and anxiety in nursing interns

The results of this study showed that anxiety was negatively correlated with PI (*p* < 0.01), consistent with the findings of Wu et al. (40). The higher the anxiety level of the nursing interns, the lower the PI. This is because the psychological development of nursing interns is still immature; facing a complex clinical setting, their experience and ability to solve problems is limited, and they are prone to negative emotions (41). Furthermore, nurses with negative psychology are more likely to accept negative information and magnify the shortcomings of the nursing profession, resulting in impaired professional values, lower PI, and burnout, which in turn leads nursing interns to leave the nursing profession, increasing the shortage of nurses (42, 43).

The results of this study showed that clinical belongingness was positively correlated with PI (*p* < 0.01), consistent with the results of Mirhosseini et al. (44). Social psychology posits that satisfying the need to belong has a positive effect on emotional patterns and behavioral responses (34). A supportive clinical environment is beneficial for increasing the sense of collective integration and emotional needs of nursing interns, which in turn encourages them to adopt a positive approach to complex clinical problems during their internships, resulting in better professional performance and professional identity. By contrast, those who lack a sense of belonging are more likely to experience anxiety and burnout (45).

### 4.3 Analysis of the mediating effect of clinical belongingness between anxiety and PI in nursing interns

Finally, by including clinical belongingness in the regression equation, we analyzed and verified the hypothesis. Clinical belongingness mediated the relationship between anxiety and PI, with a mediation effect of 40%. This result is in agreement with those of previous studies in which clinical belongingness was a protective factor for reducing anxiety and improving PI among nursing interns (46). From the perspective of positive psychology, if nursing interns feel the benefits and positive emotional experiences provided by nursing work, it can encourage them to be more active in clinical practice and have a positive mindset to deal with negative emotions, such as pressure and anxiety (47). Conversely, clinical belongingness can assist nursing interns to become more recognized for nursing work and accelerate the transformation of nursing interns into qualified medical personnel (48). Therefore, it is recommended that nursing managers and educators pay greater attention to the clinical belongingness of nursing interns from a positive psychology perspective and utilize management and teaching methods that are appropriate to the characteristics of the current nursing intern cohort to improve their sense of PI.

### 4.4 Implication for theoretical and practice

This study offers valuable theoretical foundation for nurse managers and educators in developing strategies to enhance nursing interns’ PI. On the one hand, these results theoretically testify that clinical belongingness plays a positive moderating role in the relationship between anxiety and PI. On the other hand, the results of this study are instructive for improving and enhancing the PI of nursing interns. Therefore, nurse managers and educators should pay attention to nursing interns with high levels of anxiety, low PI, and low clinical belongingness. In addition, we suggest that nursing managers and educators demonstrate caring behaviors toward patients and colleagues, setting an example for team. Regular heart-to-heart talks with nursing interns are conducted to identify and assist in solving their clinical practice difficulties, and to help them establish a correct outlook and cognition of nursing the profession, which has far-reaching implications for the overall career development of nursing interns and the quality of nursing care.

### 4.5 Limitations of this study

Several limitations need to be acknowledged. Due to time and manpower constraints, this cross-sectional survey was only conducted in Sichuan Province, China, which may limit the extension of these results to other regions. Therefore, more large-scale and multi-center studies are required to improve the generalization of these findings in various geographies. Second, this study used a cross-sectional survey, and it is impossible to identify the order of clinical belongingness, PI, and anxiety. Therefore, variables may have a reverse causal relationship, longitudinal studies will be needed in the future to explore the true causal relationship. Third, the influence path of anxiety on professional identity is multifaceted, clinical belongingness provides only a component of the mediating effect, consequently, there may be additional potential mediating effects that have not yet been identified. Therefore, qualitative interviews should be considered to collect more data to investigate potential mediating factors. Four, data were collected through online self-reporting (e.g., BES-CPE, PIQNS, SAS) may recall and response bias, potentially affecting data accuracy. Future research could incorporate face-to-face qualitative interview data or utilize objective measurement instruments to enhance data accuracy and reliability.

## 5 Conclusion

This study bridges the gap in the literature on the relationship between clinical belongingness, PI, and anxiety, and provides a new theoretical perspective for future nursing education and practice. In the context of global healthcare resource constraints, nursing educators and clinical practice managers must change traditional management and teaching concepts, offer appropriate tolerance and praise to nursing interns, reduce criticism and blame, further meet their needs for love and belonging, assist in better integration into the collective, reduce the impact of the clinical environment and professional bias, and stimulate learning motivation and work enthusiasm, thus improving PI and promoting the stability and development of nursing careers.

## Data Availability

The raw data supporting the conclusions of this article will be made available by the authors, without undue reservation.

## Footnotes

^1^ www.wjx.cn
